# Four-year observation of predictability and stability of small incision lenticule extraction

**DOI:** 10.1186/s12886-016-0331-0

**Published:** 2016-08-30

**Authors:** Tian Han, Ke Zheng, Yingjun Chen, Yang Gao, Li He, Xingtao Zhou

**Affiliations:** 1 Department of Ophthalmology and Vision Science, Eye and ENT Hospital of Fudan University, No.19 Baoqing Road, Xuhui District Shanghai, China; 2 Aier School of Ophthalmology, Central South University, Changsha, China; 3 Department of Ophthalmology, UruTmqi Eye and ENT Hospital, No.387 Zhongshan Road, Urumqi, Xinjiang China

**Keywords:** Small incision lenticule extraction (SMILE), Aberrations, Quality of life, Quality of Life Impact of Refractive Correction (QIRC) questionnaire

## Abstract

**Background:**

To investigate long-term refractive outcomes, wavefront aberrations and quality of life after small incision lenticule extraction (SMILE) for moderate to high myopia.

**Methods:**

A total of 26 patients (47 eyes) with preoperative mean spherical equivalent (SE) of -6.30 ± 1.47 diopters (D) who underwent SMILE were recruited. The measurements included uncorrected distance visual acuity (UDVA), corrected distance visual acuity (CDVA), manifest refraction, wavefront aberrations, and quality of life.

**Results:**

At 4 years postoperatively, UDVA was better than or equal to 20/20 in 92 % of eyes. The efficacy index was 1.07 ± 0.16. 89 % of eyes were within ± 0.5 D of the intended refractive target. No eye lost any Snellen lines. The safety index was 1.16 ± 0.14. No significant changes of SE occurred among postoperative follow-ups at months 1, 3, 6 and years 1, 2, 4 (*P* > 0.05, Scheffe test). Higher-order aberrations, coma, spherical aberration and higher-order astigmatism increased postoperatively, and no significant changes of aberrations were detected among the 1-month, 6-month or 4-year follow-ups postoperatively (37 eyes). Compared to the spectacles group, the surgery group showed a significantly higher total score on quality of life (45.71 ± 2.61 vs 39.96 ± 3.56, *P* < 0.001).

**Conclusions:**

SMILE provides a predictable and stable correction of moderate to high myopia as documented by long-term follow-up.

## Background

Small incision lenticule extraction (SMILE) was first introduced by Shah and Sekundo in 2011 [1, 2]. Compared to laser in situ keratomileusis (LASIK), SMILE is a minimally invasive and flap-less procedure. Promising clinical results have been reported [3–14]. Only a few articles on the long-term refractive outcomes after SMILE have been reported [13, 15]. Scientific evidence documenting SMILE over long-term follow-up is necessary to gain more support and wider acceptance of the procedure [16]. The aim of this study is to investigate four-year visual quality (refractive outcomes and aberrations) and quality of life outcomes after the SMILE procedure.

## Methods

### Subjects

Patients who underwent SMILE at the Refractive Surgery Center of the Department of Ophthalmology, Eye and ENT Hospital of Fudan University between January, 2011 and May, 2012 were enrolled in the prospective study. Inclusion criteria included age over 18 years, sphere of -3.00 − -9.00 diopters (D) with astigmatism up to -2.00 D, corrected distance visual acuity (CDVA) of 20/20 or better, stable refraction for 2 years, and no use of any kind of contact lenses within the previous 2 weeks. Patients with systemic diseases, a history of ocular surgery or trauma, or a history of ocular disease other than myopia or astigmatism were excluded. Among these myopia patients, those who received SMILE for both eyes were recruited in the quality-of-life study. Patients over 39 years of age were excluded as requisition of the Quality of Life Impact of Refractive Correction (QIRC) questionnaire [17].A group of individuals who wore spectacles full-time were enrolled as a control. Inclusion criteria included age 18–39 years, CDVA of 20/20 or better, sphere of -3.00 – -9.00 D with astigmatism up to -2.00 D, stable refraction for 2 years, use of spectacles for more than 4 years, and no other ophthalmic problems.

This study followed the tenets of the Declaration of Helsinki and was approved by the ethics committee of the Eye and ENT Hospital of Fudan University. Informed written consent was obtained from all participants.

### Procedures

The same surgeon (XTZ) performed all the SMILE procedures. The VisuMax femtosecond laser system (Carl Zeiss Meditec, Jena, Germany) was used with a repetition rate of 500 kHz and pulse energy of 130 nJ. The lenticule diameter was set at 6.5–6.7 mm and the stromal cap was completed at a 100-μm depth with a diameter of 7.5 mm. A 90-degree single side cut with a length of 2.0–4.0 mm was created during the procedure. After surgery, topical levofloxacin, 0.1 % fluorometholone solution, and non-preserved artificial tears (carboxymethylcellulose sodium eye drops; Allergan, Inc., Irvine, CA) were used.

### Measurements

The outcome measures included uncorrected distance visual acuity (UDVA), CDVA, manifest refraction, and wavefront aberrations. Routine examinations, like slit-lamp examination, rotating Scheimpflug camera imaging (Pentacam, Oculus GmbH), were also preformed.

Postoperative time points included 1, 3, 6 months and 1, 2, 4 years postoperatively.

Wavefront aberrations were measured with a Hartmann–Shack WASCA aberrometer (Carl Zeiss Meditec AG) with a 6.0 mm pupil using sixth order Zernike polynomials. The root mean square (RMS) of higher-order aberrations (HOAs), spherical aberration, coma, higher-order astigmatism, trefoil and tetrafoil was calculated. Only measurements in eyes with a pupil of 6.0 mm or larger were included. Thus, the aberration measurements of thirty-seven eyes for 1, 6 months and 4 years postoperatively were collected.

The Chinese version of the QIRC questionnaire was completed by Xu Congyi et al*.* [18] and showed favorable repeatability and validity. The QIRC questionnaire was used to assess the quality of life of the spectacles group and the surgery group at the last follow-up.

### Data analysis

All statistical analysis was performed using the Statistical Package for Social Sciences (SPSS, Version 20) (IBM, Armonk, NY, USA). The Kolmogorov–Smirnov test was used to test for normality. Non-normally distributed data were described as the mean, medium, and interquartile range (IQR). One-way analysis of variance (ANOVA) was used for the analysis of changes during the time course, with Tukey test and Scheffe test for multiple comparisons. When variables could not meet the condition of ANOVA, the Wilcoxon signed-rank test was used for paired data and the Mann-Whitney U test was used for unpaired data. For proportions, Fisher’s exact test was used. For all tests, a *P* < 0.05 was defined as statistically significant.

## Results

The preoperative mean age of the patients was 29.02 ± 7.23 years (range: 19–44 years) and mean spherical equivalent (SE) was -6.30 ± 1.47 D (range: -3.50 – -8.75 D). The follow-up was 46.43 ± 2.33 months (range: 43–58 months). None of the 47 eyes showed signs of ectasia.

### Refractive outcomes

**Fig. 1:**
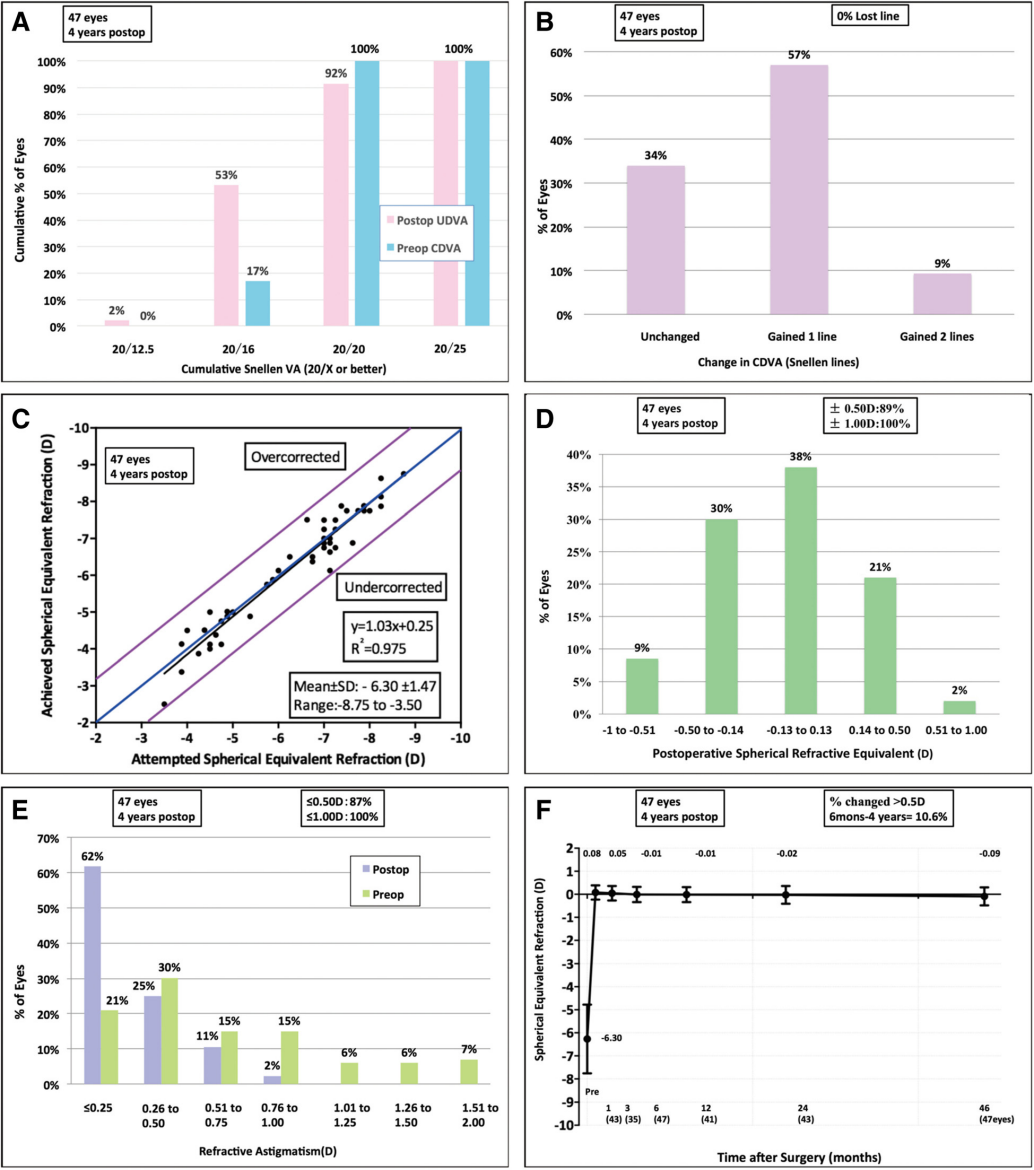
Refractive outcomes at 4 years postoperatively for 47 eyes with moderate to high myopia treated with SMILE. UDVA = uncorrected distance visual acuity; CDVA = corrected distance visual acuity; D = diopters; Postop = postoperative; Preop = preoperative

Refractive outcomes pre-operatively and at postoperative follow-ups at 6 months and 4 years are summarized in Table 1 and Fig. 1. At the 4-year follow-up, UDVA was better than or equal to 20/20 in 92 % of eyes and 20/16 in 53 %. The efficacy index was 1.07 ± 0.16. 89 % of eyes were within ± 0.5 D of the intended refractive target. No significant changes of SE occurred among postoperative follow-ups at months 1, 3, 6 and years 1, 2, 4 (*P* > 0.05, Scheffe test). No eye lost any Snellen lines and 9 % showed an increase of 2 lines. The safety index was 1.16 ± 0.14.

**Table 1:** Refractive outcomes during the follow-up period

Variables	Preoperative	6 Months postoperative	4 Years postoperative	P (Preop to 4 years postop)	P (6 Months to 4 years postop)
LogMAR UDVA					
Mean ± SD		-0.05 ± 0.06	-0.04 ± 0.06	-	0.094
Medium(IQR)	-	-0.08(-0.08 to 0)	-0.08(-0.08 to 0)		
LogMAR CDVA					
Mean	-0.01 ± 0.03	-0.08 ± 0.06	-0.08 ± 0.06	<0.001	0.570
Medium(IQR)	0(0 to 0)	-0.08(-0.08 to -0.08)	-0.08(-0.08 to -0.08)		
Sphere (D)					
Mean ± SD	-5.94 ± 1.45	0.14 ± 0.35	0.05 ± 0.41	<0.001	0.278
Range	-8.50 to -3.25	-0.50 to 1.25	-1.00 to 1.25		
Cylinder (D)					
Mean ± SD	-0.73 ± 0.49	-0.29 ± 0.27	-0.28 ± 0.29	<0.001	0.747
Medium(IQR)	-0.50(-1.00 to 0)	-0.25(-0.50 to 0)	-0.25(-0.50 to 0)		
SE					
Mean ± SD	-6.30 ± 1.47	-0.01 ± 0.33	-0.09 ± 0.39	<0.001	0.929
Range	-8.75 to -3.50	-0.75 to 0.88	-1.00 to 0.88		
The efficacy index					
Mean ± SD	-	1.11 ± 0.17	1.07 ± 0.16	-	0.275
Medium(IQR)		1.0(1.0 to 1.2)	1.0(1.0 to 1.2)		
The safety index					
Mean ± SD	-	1.17 ± 0.15	1.16 ± 0.14	-	0.622
Medium(IQR)	-	1.2(1.0 to 1.2)	1.2(1.0 to 1.2)		

*Preop* preoperative, *Postop* postoperative, *UDVA* uncorrected distance visual acuity, *IQR* interquartile range, *CDVA* corrected distance visual acuity, *D* diopters, *SE* spherical equivalent

### Wavefront aberrations

**Fig. 2:**
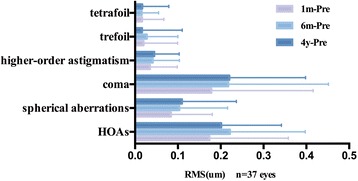
Changes of aberrations between time points after the SMILE procedure. RMS = root mean square; HOAs = higher-order aberrations; 1 m-Pre = aberrations at 1 month minus preoperative aberrations; 3 m-Pre = aberrations at 3 months minus preoperative aberrations; 4y-Pre = aberrations at 4 years minus preoperative aberrations

Aberrations outcomes are summarized in Table 2. HOAs, coma, and higher-order astigmatism increased since 1 month postoperatively (Tukey test, *P* < 0.01). Moreover, significant differences of spherical aberration were found between preoperative values and values at 6 months, 4 years postoperatively (Tukey test, *P* < 0.05). Among these aberrations, postoperative coma showed the greatest increase after the surgery (Fig. 2). No significant differences of HOAs, spherical aberration, coma, higher-order astigmatism, trefoil and tetrafoil were detected among the 1-month, 6-month or 4-year follow-ups postoperatively (Tukey test, *P* > 0.05).

**Table 2:** Time course of aberrations after SMILE

Variables	Preoperative	1 Month postoperative	6 Months postoperative	4 Years postoperative	*P*
HOAs (μm)	0.31 ± 0.12	0.48 ± 0.16	0.53 ± 0.17	0.51 ± 0.11	<0.001
Spherical aberration (μm)	0.07 ± 0.15	0.15 ± 0.13	0.17 ± 0.14	0.18 ± 0.14	0.003
Coma (μm)	0.18 ± 0.13	0.36 ± 0.23	0.40 ± 0.23	0.40 ± 0.15	<0.001
Higher-order astigmatism (μm)	0.05 ± 0.03	0.09 ± 0.06	0.09 ± 0.05	0.10 ± 0.06	0.001
Trefoil (μm)	0.14 ± 0.08	0.16 ± 0.08	0.16 ± 0.08	0.16 ± 0.09	0.693
Tetrafoil (μm)	0.06 ± 0.03	0.08 ± 0.05	0.08 ± 0.04	0.08 ± 0.06	0.294

*HOAs* higher-order aberrations

### Quality of life evaluation

No significant difference in characteristics was found between the surgery group and the spectacle group (Table 3). There are 19 subjects in the surgery group and 54 in the spectacle group, respectively. Compared to the spectacles group, the surgery group showed a significantly higher score in total score (45.71 ± 2.61 vs 39.96 ± 3.56, *P* < 0.001), the visual function, 4 of 5 convenience issues, both of the economic concerns, and 1 of 7 well-being measurements (Table 4). There were no significant differences between responses regarding symptoms, 1 of 5 convenience concerns, any of 4 health concerns, or 6 of 7 well-being measurements.

**Table 3:** Characteristics of the surgery and spectacles groups

Characteristics	Surgery group	Spectacles group	*P*
Sample Size (n)	19	54	-
Sex (M/F)	6/13	24/30	0.241
Age	31.16 ± 5.39	30.04 ± 4.76	0.521
Myopia level (the worse eye, SE: -3.00 to -6.00D/<-6.00D)	6/13	22/32	0.336
Are you satisfied with your vision? (satisfied/unsatisfied)	19/0	51/3	0.399
How would you characterize your health? (good/excellent)	10/9	28/26	0.583
CDVA for the worse eye ≥ 20/20	19/0	54/0	-

*D* diopters, *SE* spherical equivalent, *CDVA* corrected distance visual acuity

**Table 4:** Differences in QIRC questionnaire items between surgery and spectacles groups

Variables	Surgery group				Spectacles group				*P*
	Mean	SD	Medium	IQR	Mean	SD	Medium	IQR	
1. Total score	45.71	2.61	45.36	43.73 to 47.51	39.96	3.56	39.42	37.11 to 41.65	<0.001
2. How much difficulty do you have driving in glare conditions?	52.27	7.98	45.06	45.06 to 60.51	38.54	11.16	29.61	29.61 to 45.06	<0.001
3. During the past month, how often have you experienced your eyes feeling tired or strained?	46.41	8.27	49.66	34.21 to 49.66	45.08	9.31	49.66	34.21 to 49.66	0.524
4. How much trouble is not being able to use off-the-shelf (non prescription) sunglasses?	47.77	11.87	56.71	41.26 to 56.71	34.97	9.73	41.26	25.81 to 41.26	<0.001
5. How much trouble is having to think about your spectacles or contact lenses or your eyes after refractive surgery before doing things; eg, traveling, sport, going swimming?	58.93	5.79	61.37	61.37 to 61.37	41.91	12.44	45.92	30.47 to 45.92	<0.001
6. How much trouble is not being able to see when you wake up; eg, to go to the bathroom, look after a baby, see alarm clock?	56.88	5.79	59.32	59.32 to 59.32	44.44	9.94	43.87	43.87 to 43.87	<0.001
7. How much trouble is not being able to see when you are on the beach or swimming in the sea or pool, because you do these activities without spectacles or contact lenses?	57.42	11.87	63.92	48.48 to 63.92	36.66	7.93	33.03	33.03 to 33.03	<0.001
8. How much trouble is your spectacles or contact lenses when you wear them when using a gym/ doing keep-fit classes/circuit training, etc?	32.40	11.93	24.27	24.27 to 39.72	29.21	8.51	24.27	24.27 to 39.72	0.351
9. How concerned are you about the initial and ongoing cost to buy your current spectacles/ contact lenses/refractive surgery?	58.92	7.66	64.61	49.16 to 64.61	49.16	12.73	49.16	33.71 to 64.61	0.004
10. How concerned are you about the cost of unscheduled maintenance of your spectacles/ contact lenses/refractive surgery; eg, breakage, loss, new eye problems?	47.61	13.87	45.18	29.73 to 60.62	38.88	12.19	29.73	29.73 to 45.18	0.015
11. How concerned are you about having to increasingly rely on your spectacles or contact lenses since you started to wear them?	42.69	13.00	34.56	34.56 to 50.01	47.72	13.22	50.01	34.56 to 65.46	0.128
12. How concerned are you about your vision not being as good as it could be?	38.31	8.68	34.24	34.24 to 32.24	38.82	9.31	34.24	34.24 to 34.24	0.889
How concerned are you about medical complications from your choice of optical correction (spectacles, contact lenses and/or refractive surgery)?	34.28	10.57	28.59	28.59 to 44.04	38.89	11.23	44.04	28.59 to 44.04	0.084
13. How concerned are you about eye protection from ultraviolet (UV) radiation?	39.79	8.68	35.72	35.72 to 35.72	43.73	11.52	35.72	35.72 to 51.17	0.182
14. During the past month, how much of the time have you felt that you have looked your best?	39.75	12.36	45.52	28.25 to 45.52	38.11	12.97	28.25	28.25 to 45.52	0.536
15. During the past month, how much of the time have you felt that you think others see you the way you would like them to (eg, intelligent, sophisticated, successful, cool, etc)?	47.17	5.45	48.99	48.99 to 48.99	43.01	11.01	48.99	31.72 to 48.99	0.079
16. During the past month, how much of the time have you felt complimented/flattered?	51.51	11.34	54.55	37.28 to 54.55	48.50	12.01	54.55	37.28 to 54.55	0.304
17. During the past month, how much of the time have you felt confident?	44.87	9.63	42.67	42.67 to 57.94	39.10	11.98	42.67	25.40 to 42.67	0.063
18. During the past month, how much of the time have you felt happy?	46.04	7.75	39.61	39.61 to 54.88	38.60	10.51	39.61	39.61 to 39.61	0.008
19. During the past month, how much of the time have you felt able to do the things you want to do?	30.65	6.71	31.66	31.66 to 31.66	27.59	15.93	31.66	14.39 to 31.66	0.078
20. During the past month, how much of the time have you felt eager to try new things?	42.61	9.10	41.22	41.22 to 41.22	38.76	17.08	41.22	23.95 to 41.22	0.067

*QIRC* Quality of Life Impact of Refractive Correction, *IQR* interquartile range

## Discussion

SMILE has proven to be an effective, predictable and safe procedure since Shah and Sekundo first introduced it five years ago [3–12]. In this study, we report the long-term observation of visual quality (refractive outcomes and aberrations) and quality of life outcomes in SMILE patients up to 58 months.

In this study, UDVA was better than or equal to 20/20 in 92 % of eyes and 20/16 in 53 %. No eye lost any line of CDVA and 9 % showed an increase of 2 lines. The efficacy and safety index were 1.07 ± 0.16 and 1.16 ± 0.14, respectively. Pedersen et al. showed 72 % eyes with 20/20 or better UDVA in 57 eyes of high myopia targeted for emmetropia at 3 years after SMILE procedure [15]. The discrepancy of UDVA results might due to the different study subjects. Pedersen et al. showed the refractive outcomes of patients with high myopia (92 % of eyes more than -6.00D), while in this study, we studied the outcomes after SMILE for moderate to high myopia (59 % of eyes more than -6.00D). Kim et al. [3] observed that at 12 months postoperatively, 93.1 % of the eyes in the mild- to moderate-myopia group and 76.8 % of eyes in high myopia group had UDVA of 20/20 or better (*P* < 0.05). Blum et al. showed the efficacy index and safety index of the worldwide first 41 eyes treated using SMILE at the 5-year follow-up was 0.9 and 1.2, respectively [13]. They performed SMILE with the old type VisuMax at a repetition rate of 200 kHz with a typical pulse energy <300 nJ, while we used the new type VisuMax at a repetition rate of 500 kHz. A 500 kHz VisuMax caused less tissue damage and resulted in better outcomes. These results indicate the favorable efficacy and safety of the SMILE procedure.

Considering long-term predictability and stability, 89 % of eyes were within 0.5 D and 100 % were within 1.0 D of the intended refractive target. In the study by Pedersen IB et al., these values were 78 % and 90 % respectively [15]. Blum et al. observed that 48.2 % of eyes were within 0.5 D and 78.6 % were within 1.0 D [13]. In our study, no significant changes in SE occurred between postoperative follow-ups, although SE was decreased from -0.01 ± 0.33 D at 6 months to -0.09 ± 0.39 D at four years after SMILE. No significant changes of SE occurred were also reported by Pedersen et al. and Blum et al. [13, 15]. These results demonstrate the predictability and stability of refractive outcomes after SMILE with 4 years of follow up. In terms of other refractive surgeries, several refractive results after LASIK [19–21] and photorefractive keratectomy (PRK) [22] showed a significant decline in SE over about ten years after the surgery, especially for high corrections and young patients. Therefore, it is necessary to investigate the stability of SMILE in the long term.

HOAs play an important role in retinal image quality. Similar to previous studies [1, 2, 8, 23], total HOAs, coma, spherical aberration and higher-order astigmatism increased postoperatively. Among these aberrations, postoperative coma was most affected and remained stable at all follow-up time points. The induced coma might be associated with decentration and special efforts should be made to minimize induced coma clinically [24]. In addition, no significant changes of aberrations were detected among the 1-month, 6-month, or 4-year follow-ups postoperatively. However, this is in contrast to a previous study using the Pentacam to analyze the anterior, posterior and total corneal aberrations, in which HOAs and spherical aberrations significantly decreased from 3 months to 3 years after SMILE [15]. In this study, we used the Hartmann–Shack WASCA aberrometer which measures the whole-eye wavefront aberrations. Different measurements might result in the different outcomes and the long-term changes of aberrations on SMILE still need further discussion.

Quality of life metrics assesses the changes in physical, functional, mental and social health in individuals. The comparison of quality of life between common treatments to myopia is helpful to evaluate the benefits of the refractive surgery. The QIRC questionnaire targets patients with refractive correction by spectacles, contact lenses and refractive surgery, and is rigorously developed using both conventional techniques and Rasch analysis [17]. The QIRC questionnaire has been used to measure differences between patients with correction by LASIK and other two modes of refractive correction [25, 26]. In this study, long-term quality of life outcomes on SMILE patients were studied. The total score of the QIRC questionnaire was significantly higher in the surgery group than the spectacles group. Compared to patients with refractive correction by spectacles, patients who underwent SMILE showed better refractive error-related quality of life and SMILE brings both economic benefits and convenience to individuals with moderate to high myopia.

A major weakness of this article is the relatively small sample size. It may limit the precision of the results. Further randomized and multi-centered studies with a larger sample size were of clinical significance.

## Conclusions

In conclusion, SMILE provides a predictable and stable correction of moderate to high myopia in the long-term follow-up. Patients who underwent SMILE showed better quality of life compared to individuals who wore spectacles.

## Abbreviations

ANOVA: Analysis of variance
CDVA: Corrected distance visual acuity
D: Diopters
HOAs: Higher-order aberrations
IQR: Interquartile range
LASIK: Laser in situ keratomileusis
PRK: Photorefractive keratectomy
QIRC: Quality of life impact of refractive correction
RMS: Root mean square
SE: Spherical equivalent
SMILE: Small incision lenticule extraction
SPSS: Statistical package for social sciences
UDVA: Uncorrected distance visual acuity
